# 7′-(4-Bromo­phen­yl)-5′,6′,7′,7a’-tetra­hydro­dispiro­[indan-2,5′-pyrrolo­[1,2-*c*][1,3]thia­zole-6′,2′′-indan]-1,3,1′′-trione

**DOI:** 10.1107/S1600536811046514

**Published:** 2011-11-12

**Authors:** Ang Chee Wei, Mohamed Ashraf Ali, Yeong Keng Yoon, Ching Kheng Quah, Hoong-Kun Fun

**Affiliations:** a Institute for Research in Molecular Medicine, Universiti Sains Malaysia, 11800 USM, Penang, Malaysia; b X-ray Crystallography Unit, School of Physics, Universiti Sains Malaysia, 11800 USM, Penang, Malaysia

## Abstract

In the title compound, C_28_H_20_BrNO_3_S, the thia­zolidine, pyrrolidine and two five-membered carbocyclic rings are in envelope conformations. The bromo-bound phenyl ring forms dihedral angles of 61.97 (18) and 88.30 (17)° with the other two benzene rings. The two benzene rings form a dihedral angle of 30.3 (2)°. The mol­ecular structure features an intra­molecular C—H⋯O hydrogen bond, which generates an *S*(6) ring motif. In the crystal, mol­ecules are linked into inversion dimers by pairs of C—H⋯O hydrogen bonds.

## Related literature

For related structures and background references, see: Wei *et al.* (2011*a* ▶,*b* ▶,*c* ▶); Kumar *et al.* (2010 ▶). For hydrogen-bond motifs, see: Bernstein *et al.* (1995 ▶). For ring conformations, see: Cremer & Pople (1975 ▶). For bond-length data, see: Allen *et al.* (1987 ▶).
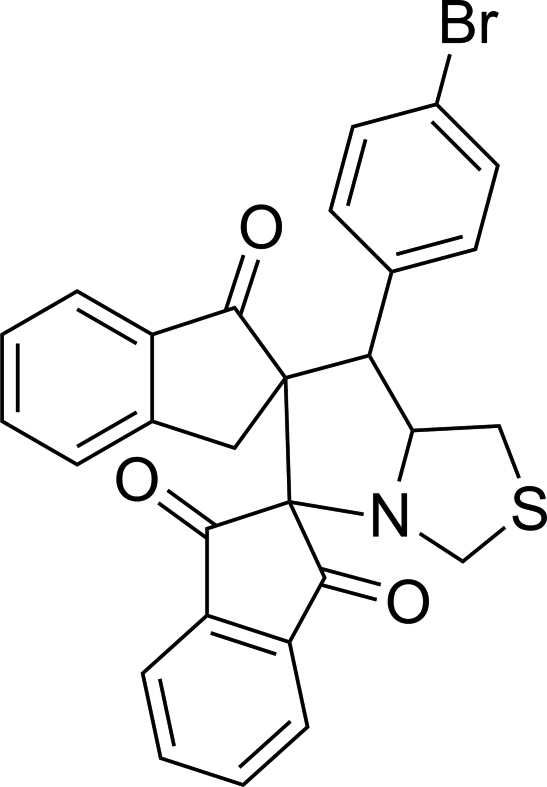


## Experimental

### 

#### Crystal data

C_28_H_20_BrNO_3_S
*M* *_r_* = 530.42
Triclinic, 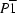

*a* = 10.2871 (11) Å
*b* = 11.1375 (12) Å
*c* = 11.5877 (13) Å
α = 115.511 (2)°
β = 90.075 (2)°
γ = 97.347 (2)°
*V* = 1186.0 (2) Å^3^
*Z* = 2
Mo *K*α radiation
μ = 1.85 mm^−1^
*T* = 296 K
0.31 × 0.13 × 0.05 mm

#### Data collection

Bruker SMART APEXII DUO CCD area-detector diffractometer
Absorption correction: multi-scan (*SADABS*; Bruker, 2009 ▶) *T*_min_ = 0.599, *T*_max_ = 0.915
17888 measured reflections
5422 independent reflections
3789 reflections with *I* > 2σ(*I*)
*R*_int_ = 0.028

#### Refinement

*R*[*F* ^2^ > 2σ(*F* ^2^)] = 0.044
*wR*(*F* ^2^) = 0.136
*S* = 1.04
5422 reflections
307 parameters
H-atom parameters constrained
Δρ_max_ = 0.76 e Å^−3^
Δρ_min_ = −0.55 e Å^−3^

### 

Data collection: *APEX2* (Bruker, 2009 ▶); cell refinement: *SAINT* (Bruker, 2009 ▶); data reduction: *SAINT*; program(s) used to solve structure: *SHELXTL* (Sheldrick, 2008 ▶); program(s) used to refine structure: *SHELXTL*; molecular graphics: *SHELXTL*; software used to prepare material for publication: *SHELXTL* and *PLATON* (Spek, 2009 ▶).

## Supplementary Material

Crystal structure: contains datablock(s) global, I. DOI: 10.1107/S1600536811046514/hg5131sup1.cif

Structure factors: contains datablock(s) I. DOI: 10.1107/S1600536811046514/hg5131Isup2.hkl

Additional supplementary materials:  crystallographic information; 3D view; checkCIF report

## Figures and Tables

**Table 1:** Hydrogen-bond geometry (Å, °)

*D*—H⋯*A*	*D*—H	H⋯*A*	*D*⋯*A*	*D*—H⋯*A*
C18—H18*B*⋯O1	0.97	2.42	3.080 (4)	125
C22—H22*A*⋯O2^i^	0.93	2.44	3.172 (4)	135

Symmetry code: (i) 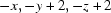.

**Fig. 1.**
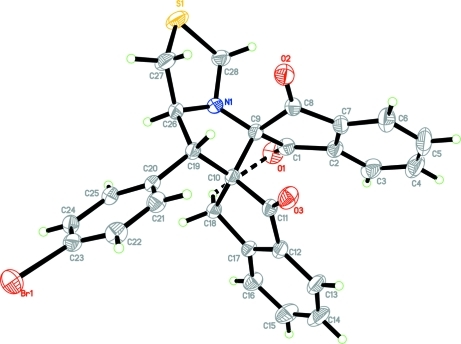
The molecular structure of the title compound showing 30% probability displacement ellipsoids for non-H atoms. Intramolecular hydrogen bond is shown as dashed line.

**Fig. 2.**
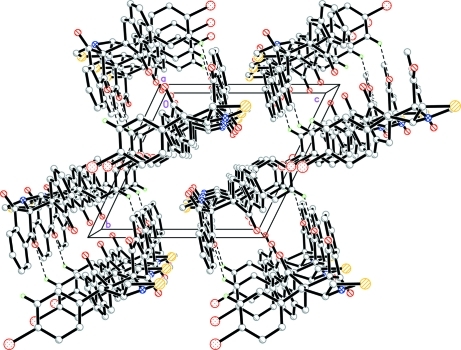
The crystal structure of the title compound, viewed along the a axis. H atoms not involved in hydrogen bonds (dashed lines) have been omitted for clarity.

## supplementary crystallographic information

### Comment

As part of our ongoing search for novel heterocyclic compounds with antitubercular activity (Wei *et al.*, 2011*a*, 2011*b*, 2011*c*), our group has synthesized the title compound as described below.

The molecular structure is shown in Fig. 1. The thiazolidine (S1/N1/C26-C28), pyrrolidine (N1/C9/C10/C19/C26) and two five-membered carbocyclic (C1/C2/C7-C9 and C10-C12/C17/C18) rings are in envelope conformations, puckering parameters (Cremer & Pople, 1975) Q = 0.373 (3) Å and φ = 31.3 (5)° with atom C27 at the flap; Q = 0.456 (3) Å and φ = 243.8 (4)° with atom C10 at the flap; Q = 0.229 (4) Å and φ = 330.0 (9)° with atom C9 at the flap; and Q = 0.168 (3) Å and φ = 171.2 (12)° with atom C10 at the flap, respectively. Bond lengths (Allen *et al.*, 1987) and angles are within normal ranges and are comparable to related structures (Kumar *et al.*, 2010; Wei *et al.*, 2011*a*,b,c). The bromo-bound phenyl (C20-C25) ring forms dihedral angles of 61.97 (18) and 88.30 (17)° with the other two phenyl (C2-C7 and C12-C17) rings, respectively. The two phenyl rings form a dihedral angle of 30.3 (2)°. The molecular structure is stabilized by an intramolecular C18–H18B···O1 hydrogen bond (Table 1), which generates an *S*(6) ring motif (Fig. 1, Bernstein *et al.*, 1995).

In the crystal (Fig. 2), molecules are linked into inversion dimers by pairs of intermolecular C22–H22A···O2 hydrogen bonds (Table 1).

### Experimental

A mixture of (*Z*)2-(4-bromobenzylidene)-2,3-dihydro-1*H*-indene-1-one (0.298gm, 0.001 mol), ninhydrin (0.178gm, 0.001 mol) and thiazolidine-4-carboxylic acid (0.266gm, 0.002 mol) (1:1:2) were dissolved in methanol (10 ml) and refluxed for 4 h. After completion of the reaction as evident from TLC, the mixture was poured into crushed ice. The precipitated solid was filtered, washed and recrystallised from petroleum ether–ethyl acetate mixture (1:1) to afford the title compound as yellow crystals. M.p. : 487 K.

### Refinement

All H atoms were positioned geometrically and refined using a riding model with C–H = 0.93-0.98 Å and *U*_iso_(H) = 1.2 *U*_eq_(C).

### Crystal data

**Table:**

C_28_H_20_BrNO_3_S	*Z* = 2
*M**_r_* = 530.42	*F*(000) = 540
Triclinic, *P*1	*D*_x_ = 1.485 Mg m^−^^3^
Hall symbol: -P 1	Mo *K*α radiation, λ = 0.71073 Å
*a* = 10.2871 (11) Å	Cell parameters from 5183 reflections
*b* = 11.1375 (12) Å	θ = 2.7–25.1°
*c* = 11.5877 (13) Å	µ = 1.85 mm^−^^1^
α = 115.511 (2)°	*T* = 296 K
β = 90.075 (2)°	Plate, yellow
γ = 97.347 (2)°	0.31 × 0.13 × 0.05 mm
*V* = 1186.0 (2) Å^3^	

### Data collection

**Table:**

Bruker SMART APEXII DUO CCD area-detector diffractometer	5422 independent reflections
Radiation source: fine-focus sealed tube	3789 reflections with *I* > 2σ(*I*)
graphite	*R*_int_ = 0.028
φ and ω scans	θ_max_ = 27.5°, θ_min_ = 2.0°
Absorption correction: multi-scan (*SADABS*; Bruker, 2009)	*h* = −13→13
*T*_min_ = 0.599, *T*_max_ = 0.915	*k* = −14→14
17888 measured reflections	*l* = −15→15

### Refinement

**Table:**

Refinement on *F*^2^	Primary atom site location: structure-invariant direct methods
Least-squares matrix: full	Secondary atom site location: difference Fourier map
*R*[*F*^2^ > 2σ(*F*^2^)] = 0.044	Hydrogen site location: inferred from neighbouring sites
*wR*(*F*^2^) = 0.136	H-atom parameters constrained
*S* = 1.04	*w* = 1/[σ^2^(*F*_o_^2^) + (0.069*P*)^2^ + 0.5361*P*] where *P* = (*F*_o_^2^ + 2*F*_c_^2^)/3
5422 reflections	(Δ/σ)_max_ = 0.001
307 parameters	Δρ_max_ = 0.76 e Å^−^^3^
0 restraints	Δρ_min_ = −0.55 e Å^−^^3^

### Special details

**Table:**

Geometry. All esds (except the esd in the dihedral angle between two l.s. planes) are estimated using the full covariance matrix. The cell esds are taken into account individually in the estimation of esds in distances, angles and torsion angles; correlations between esds in cell parameters are only used when they are defined by crystal symmetry. An approximate (isotropic) treatment of cell esds is used for estimating esds involving l.s. planes.
Refinement. Refinement of F^2^ against ALL reflections. The weighted R-factor wR and goodness of fit S are based on F^2^, conventional R-factors R are based on F, with F set to zero for negative F^2^. The threshold expression of F^2^ > 2sigma(F^2^) is used only for calculating R-factors(gt) etc. and is not relevant to the choice of reflections for refinement. R-factors based on F^2^ are statistically about twice as large as those based on F, and R- factors based on ALL data will be even larger.

### Fractional atomic coordinates and isotropic or equivalent isotropic displacement parameters (Å^2^)

**Table:**

	*x*	*y*	*z*	*U*_iso_*/*U*_eq_	
Br1	−0.23515 (4)	0.46981 (4)	1.06048 (4)	0.06794 (16)	
S1	−0.16572 (9)	0.80844 (13)	0.47197 (11)	0.0762 (3)	
O1	0.3178 (2)	0.7025 (2)	0.4532 (2)	0.0603 (6)	
O2	0.0988 (2)	1.0386 (2)	0.7289 (2)	0.0534 (5)	
O3	0.2534 (2)	0.9395 (2)	0.9235 (2)	0.0541 (6)	
N1	0.0550 (2)	0.7507 (2)	0.5431 (2)	0.0386 (5)	
C1	0.3018 (3)	0.7992 (3)	0.5499 (3)	0.0433 (6)	
C2	0.3960 (3)	0.9234 (3)	0.6201 (3)	0.0448 (7)	
C3	0.5303 (3)	0.9444 (4)	0.6109 (4)	0.0635 (9)	
H3A	0.5742	0.8755	0.5562	0.076*	
C4	0.5965 (4)	1.0703 (5)	0.6853 (5)	0.0809 (13)	
H4A	0.6871	1.0866	0.6829	0.097*	
C5	0.5297 (4)	1.1739 (5)	0.7641 (5)	0.0828 (13)	
H5A	0.5766	1.2588	0.8118	0.099*	
C6	0.3960 (4)	1.1540 (4)	0.7733 (3)	0.0614 (9)	
H6A	0.3519	1.2237	0.8264	0.074*	
C7	0.3297 (3)	1.0265 (3)	0.7008 (3)	0.0446 (7)	
C8	0.1887 (3)	0.9741 (3)	0.6930 (3)	0.0394 (6)	
C9	0.1741 (2)	0.8187 (3)	0.6216 (3)	0.0353 (6)	
C10	0.1681 (2)	0.7534 (3)	0.7189 (2)	0.0341 (5)	
C11	0.2667 (3)	0.8332 (3)	0.8357 (3)	0.0384 (6)	
C12	0.3772 (3)	0.7561 (3)	0.8172 (3)	0.0398 (6)	
C13	0.4989 (3)	0.7948 (4)	0.8868 (3)	0.0552 (8)	
H13A	0.5180	0.8777	0.9576	0.066*	
C14	0.5894 (3)	0.7062 (4)	0.8473 (4)	0.0717 (11)	
H14A	0.6717	0.7297	0.8906	0.086*	
C15	0.5583 (3)	0.5823 (4)	0.7433 (4)	0.0686 (10)	
H15A	0.6203	0.5235	0.7187	0.082*	
C16	0.4386 (3)	0.5428 (3)	0.6747 (3)	0.0529 (8)	
H16A	0.4195	0.4589	0.6052	0.064*	
C17	0.3475 (3)	0.6321 (3)	0.7127 (3)	0.0375 (6)	
C18	0.2104 (3)	0.6122 (3)	0.6556 (3)	0.0386 (6)	
H18A	0.1525	0.5488	0.6756	0.046*	
H18B	0.2099	0.5795	0.5633	0.046*	
C19	0.0224 (2)	0.7599 (3)	0.7500 (3)	0.0358 (6)	
H19A	0.0152	0.8546	0.8037	0.043*	
C20	−0.0338 (2)	0.6824 (3)	0.8229 (3)	0.0356 (6)	
C21	−0.0347 (3)	0.7498 (3)	0.9562 (3)	0.0442 (7)	
H21A	0.0024	0.8396	0.9982	0.053*	
C22	−0.0899 (3)	0.6850 (3)	1.0270 (3)	0.0487 (7)	
H22A	−0.0881	0.7301	1.1158	0.058*	
C23	−0.1472 (3)	0.5537 (3)	0.9644 (3)	0.0429 (6)	
C24	−0.1460 (3)	0.4830 (3)	0.8343 (3)	0.0409 (6)	
H24A	−0.1827	0.3930	0.7936	0.049*	
C25	−0.0886 (3)	0.5483 (3)	0.7640 (3)	0.0381 (6)	
H25A	−0.0871	0.5009	0.6756	0.046*	
C26	−0.0479 (3)	0.7231 (3)	0.6201 (3)	0.0414 (6)	
H26A	−0.0829	0.6272	0.5800	0.050*	
C27	−0.1579 (3)	0.8053 (4)	0.6265 (4)	0.0647 (10)	
H27A	−0.1378	0.8957	0.6947	0.078*	
H27B	−0.2406	0.7633	0.6414	0.078*	
C28	0.0078 (3)	0.8013 (3)	0.4550 (3)	0.0470 (7)	
H28A	0.0233	0.7420	0.3675	0.056*	
H28B	0.0544	0.8902	0.4755	0.056*	

### Atomic displacement parameters (Å^2^)

**Table:**

	*U*^11^	*U*^22^	*U*^33^	*U*^12^	*U*^13^	*U*^23^
Br1	0.0633 (3)	0.0906 (3)	0.0682 (3)	0.00456 (19)	0.01299 (18)	0.0533 (2)
S1	0.0400 (5)	0.1299 (9)	0.0944 (7)	0.0093 (5)	−0.0060 (4)	0.0830 (7)
O1	0.0624 (15)	0.0585 (14)	0.0492 (13)	0.0145 (11)	0.0144 (11)	0.0115 (12)
O2	0.0492 (13)	0.0408 (11)	0.0637 (14)	0.0105 (10)	0.0092 (10)	0.0156 (10)
O3	0.0513 (13)	0.0450 (12)	0.0475 (12)	0.0035 (10)	−0.0079 (10)	0.0036 (10)
N1	0.0365 (12)	0.0402 (12)	0.0399 (12)	0.0020 (10)	−0.0049 (9)	0.0192 (10)
C1	0.0411 (16)	0.0490 (17)	0.0414 (16)	0.0090 (13)	0.0064 (12)	0.0204 (14)
C2	0.0369 (15)	0.0549 (18)	0.0472 (16)	0.0011 (13)	0.0006 (12)	0.0278 (14)
C3	0.0393 (18)	0.090 (3)	0.072 (2)	0.0106 (18)	0.0107 (16)	0.045 (2)
C4	0.040 (2)	0.116 (4)	0.096 (3)	−0.018 (2)	−0.011 (2)	0.063 (3)
C5	0.066 (3)	0.079 (3)	0.089 (3)	−0.035 (2)	−0.017 (2)	0.035 (2)
C6	0.061 (2)	0.0531 (19)	0.058 (2)	−0.0120 (16)	−0.0053 (16)	0.0178 (16)
C7	0.0436 (16)	0.0438 (16)	0.0460 (16)	−0.0044 (13)	−0.0024 (13)	0.0222 (14)
C8	0.0393 (15)	0.0374 (14)	0.0387 (14)	0.0034 (12)	0.0023 (12)	0.0146 (12)
C9	0.0320 (13)	0.0341 (13)	0.0376 (14)	0.0034 (10)	0.0018 (11)	0.0139 (11)
C10	0.0291 (13)	0.0329 (13)	0.0367 (14)	0.0004 (10)	−0.0031 (10)	0.0128 (11)
C11	0.0377 (15)	0.0356 (14)	0.0361 (14)	−0.0047 (11)	−0.0079 (11)	0.0130 (12)
C12	0.0316 (14)	0.0457 (16)	0.0398 (15)	−0.0003 (11)	−0.0017 (11)	0.0180 (13)
C13	0.0404 (17)	0.062 (2)	0.0557 (19)	−0.0019 (15)	−0.0151 (14)	0.0210 (16)
C14	0.0359 (18)	0.098 (3)	0.084 (3)	0.0115 (18)	−0.0127 (17)	0.041 (2)
C15	0.0449 (19)	0.082 (3)	0.080 (3)	0.0274 (18)	0.0018 (18)	0.031 (2)
C16	0.0449 (17)	0.0545 (19)	0.0543 (19)	0.0165 (14)	0.0028 (14)	0.0164 (15)
C17	0.0292 (13)	0.0425 (15)	0.0416 (15)	0.0038 (11)	0.0000 (11)	0.0193 (12)
C18	0.0345 (14)	0.0357 (14)	0.0415 (15)	0.0006 (11)	−0.0058 (11)	0.0143 (12)
C19	0.0306 (13)	0.0335 (13)	0.0403 (14)	0.0015 (10)	−0.0013 (11)	0.0142 (12)
C20	0.0267 (13)	0.0398 (14)	0.0413 (15)	0.0041 (10)	−0.0007 (10)	0.0187 (12)
C21	0.0433 (16)	0.0401 (15)	0.0409 (16)	0.0037 (12)	−0.0005 (12)	0.0104 (13)
C22	0.0507 (18)	0.0543 (18)	0.0356 (15)	0.0088 (14)	0.0025 (13)	0.0140 (14)
C23	0.0340 (14)	0.0562 (18)	0.0476 (16)	0.0092 (13)	0.0067 (12)	0.0303 (14)
C24	0.0369 (15)	0.0392 (15)	0.0448 (16)	0.0013 (12)	0.0003 (12)	0.0178 (13)
C25	0.0342 (14)	0.0402 (15)	0.0361 (14)	0.0030 (11)	0.0004 (11)	0.0136 (12)
C26	0.0363 (15)	0.0441 (15)	0.0459 (16)	−0.0039 (12)	−0.0080 (12)	0.0242 (13)
C27	0.0330 (16)	0.107 (3)	0.082 (3)	0.0131 (17)	0.0047 (15)	0.066 (2)
C28	0.0449 (16)	0.0556 (18)	0.0474 (17)	0.0091 (14)	−0.0008 (13)	0.0284 (15)

### Geometric parameters (Å, °)

**Table:**

Br1—C23	1.901 (3)	C13—C14	1.377 (5)
S1—C28	1.805 (3)	C13—H13A	0.9300
S1—C27	1.808 (4)	C14—C15	1.383 (6)
O1—C1	1.204 (4)	C14—H14A	0.9300
O2—C8	1.208 (3)	C15—C16	1.378 (5)
O3—C11	1.209 (3)	C15—H15A	0.9300
N1—C9	1.439 (3)	C16—C17	1.387 (4)
N1—C28	1.469 (4)	C16—H16A	0.9300
N1—C26	1.473 (4)	C17—C18	1.505 (4)
C1—C2	1.483 (4)	C18—H18A	0.9700
C1—C9	1.543 (4)	C18—H18B	0.9700
C2—C3	1.383 (4)	C19—C20	1.514 (4)
C2—C7	1.391 (4)	C19—C26	1.531 (4)
C3—C4	1.374 (6)	C19—H19A	0.9800
C3—H3A	0.9300	C20—C25	1.386 (4)
C4—C5	1.390 (7)	C20—C21	1.397 (4)
C4—H4A	0.9300	C21—C22	1.387 (4)
C5—C6	1.377 (6)	C21—H21A	0.9300
C5—H5A	0.9300	C22—C23	1.370 (4)
C6—C7	1.383 (4)	C22—H22A	0.9300
C6—H6A	0.9300	C23—C24	1.368 (4)
C7—C8	1.480 (4)	C24—C25	1.395 (4)
C8—C9	1.551 (4)	C24—H24A	0.9300
C9—C10	1.580 (4)	C25—H25A	0.9300
C10—C11	1.543 (4)	C26—C27	1.526 (4)
C10—C18	1.544 (4)	C26—H26A	0.9800
C10—C19	1.546 (4)	C27—H27A	0.9700
C11—C12	1.471 (4)	C27—H27B	0.9700
C12—C17	1.385 (4)	C28—H28A	0.9700
C12—C13	1.402 (4)	C28—H28B	0.9700
			
C28—S1—C27	92.49 (14)	C15—C16—C17	117.9 (3)
C9—N1—C28	119.0 (2)	C15—C16—H16A	121.1
C9—N1—C26	110.4 (2)	C17—C16—H16A	121.1
C28—N1—C26	113.6 (2)	C12—C17—C16	120.3 (3)
O1—C1—C2	127.6 (3)	C12—C17—C18	111.6 (2)
O1—C1—C9	125.5 (3)	C16—C17—C18	128.1 (3)
C2—C1—C9	106.8 (2)	C17—C18—C10	104.2 (2)
C3—C2—C7	121.3 (3)	C17—C18—H18A	110.9
C3—C2—C1	128.7 (3)	C10—C18—H18A	110.9
C7—C2—C1	110.0 (2)	C17—C18—H18B	110.9
C4—C3—C2	117.7 (4)	C10—C18—H18B	110.9
C4—C3—H3A	121.1	H18A—C18—H18B	108.9
C2—C3—H3A	121.1	C20—C19—C26	116.1 (2)
C3—C4—C5	120.9 (3)	C20—C19—C10	116.8 (2)
C3—C4—H4A	119.6	C26—C19—C10	103.4 (2)
C5—C4—H4A	119.6	C20—C19—H19A	106.6
C6—C5—C4	121.7 (4)	C26—C19—H19A	106.6
C6—C5—H5A	119.1	C10—C19—H19A	106.6
C4—C5—H5A	119.1	C25—C20—C21	117.8 (3)
C5—C6—C7	117.4 (4)	C25—C20—C19	123.4 (2)
C5—C6—H6A	121.3	C21—C20—C19	118.8 (2)
C7—C6—H6A	121.3	C22—C21—C20	121.1 (3)
C6—C7—C2	120.9 (3)	C22—C21—H21A	119.5
C6—C7—C8	129.8 (3)	C20—C21—H21A	119.5
C2—C7—C8	109.3 (2)	C23—C22—C21	119.2 (3)
O2—C8—C7	127.3 (3)	C23—C22—H22A	120.4
O2—C8—C9	125.0 (2)	C21—C22—H22A	120.4
C7—C8—C9	107.6 (2)	C24—C23—C22	121.7 (3)
N1—C9—C1	115.4 (2)	C24—C23—Br1	119.3 (2)
N1—C9—C8	117.0 (2)	C22—C23—Br1	119.0 (2)
C1—C9—C8	101.0 (2)	C23—C24—C25	118.8 (3)
N1—C9—C10	101.06 (19)	C23—C24—H24A	120.6
C1—C9—C10	111.5 (2)	C25—C24—H24A	120.6
C8—C9—C10	111.2 (2)	C20—C25—C24	121.5 (3)
C11—C10—C18	104.5 (2)	C20—C25—H25A	119.3
C11—C10—C19	114.4 (2)	C24—C25—H25A	119.3
C18—C10—C19	116.7 (2)	N1—C26—C27	108.7 (2)
C11—C10—C9	111.9 (2)	N1—C26—C19	104.9 (2)
C18—C10—C9	110.4 (2)	C27—C26—C19	114.3 (3)
C19—C10—C9	99.1 (2)	N1—C26—H26A	109.6
O3—C11—C12	128.0 (2)	C27—C26—H26A	109.6
O3—C11—C10	125.1 (3)	C19—C26—H26A	109.6
C12—C11—C10	107.0 (2)	C26—C27—S1	104.9 (2)
C17—C12—C13	121.3 (3)	C26—C27—H27A	110.8
C17—C12—C11	109.8 (2)	S1—C27—H27A	110.8
C13—C12—C11	128.8 (3)	C26—C27—H27B	110.8
C14—C13—C12	118.0 (3)	S1—C27—H27B	110.8
C14—C13—H13A	121.0	H27A—C27—H27B	108.9
C12—C13—H13A	121.0	N1—C28—S1	108.2 (2)
C13—C14—C15	120.1 (3)	N1—C28—H28A	110.1
C13—C14—H14A	119.9	S1—C28—H28A	110.1
C15—C14—H14A	119.9	N1—C28—H28B	110.1
C16—C15—C14	122.4 (3)	S1—C28—H28B	110.1
C16—C15—H15A	118.8	H28A—C28—H28B	108.4
C14—C15—H15A	118.8		
			
O1—C1—C2—C3	−17.4 (5)	C10—C11—C12—C17	−8.1 (3)
C9—C1—C2—C3	164.9 (3)	O3—C11—C12—C13	−9.1 (5)
O1—C1—C2—C7	161.0 (3)	C10—C11—C12—C13	170.2 (3)
C9—C1—C2—C7	−16.6 (3)	C17—C12—C13—C14	0.8 (5)
C7—C2—C3—C4	0.7 (5)	C11—C12—C13—C14	−177.2 (3)
C1—C2—C3—C4	179.0 (3)	C12—C13—C14—C15	−1.3 (6)
C2—C3—C4—C5	−1.9 (6)	C13—C14—C15—C16	0.8 (7)
C3—C4—C5—C6	1.7 (7)	C14—C15—C16—C17	0.2 (6)
C4—C5—C6—C7	−0.1 (6)	C13—C12—C17—C16	0.2 (4)
C5—C6—C7—C2	−1.1 (5)	C11—C12—C17—C16	178.5 (3)
C5—C6—C7—C8	178.3 (3)	C13—C12—C17—C18	178.6 (3)
C3—C2—C7—C6	0.8 (5)	C11—C12—C17—C18	−3.0 (3)
C1—C2—C7—C6	−177.8 (3)	C15—C16—C17—C12	−0.7 (5)
C3—C2—C7—C8	−178.7 (3)	C15—C16—C17—C18	−178.8 (3)
C1—C2—C7—C8	2.7 (3)	C12—C17—C18—C10	12.7 (3)
C6—C7—C8—O2	16.4 (5)	C16—C17—C18—C10	−169.0 (3)
C2—C7—C8—O2	−164.2 (3)	C11—C10—C18—C17	−16.5 (3)
C6—C7—C8—C9	−167.2 (3)	C19—C10—C18—C17	−143.9 (2)
C2—C7—C8—C9	12.2 (3)	C9—C10—C18—C17	104.0 (2)
C28—N1—C9—C1	−71.8 (3)	C11—C10—C19—C20	−72.1 (3)
C26—N1—C9—C1	154.2 (2)	C18—C10—C19—C20	50.3 (3)
C28—N1—C9—C8	46.8 (3)	C9—C10—C19—C20	168.7 (2)
C26—N1—C9—C8	−87.2 (3)	C11—C10—C19—C26	159.1 (2)
C28—N1—C9—C10	167.7 (2)	C18—C10—C19—C26	−78.6 (3)
C26—N1—C9—C10	33.7 (3)	C9—C10—C19—C26	39.9 (2)
O1—C1—C9—N1	−28.3 (4)	C26—C19—C20—C25	35.0 (4)
C2—C1—C9—N1	149.5 (2)	C10—C19—C20—C25	−87.5 (3)
O1—C1—C9—C8	−155.4 (3)	C26—C19—C20—C21	−142.9 (3)
C2—C1—C9—C8	22.3 (3)	C10—C19—C20—C21	94.7 (3)
O1—C1—C9—C10	86.3 (3)	C25—C20—C21—C22	−0.7 (4)
C2—C1—C9—C10	−96.0 (3)	C19—C20—C21—C22	177.3 (3)
O2—C8—C9—N1	29.4 (4)	C20—C21—C22—C23	−1.6 (5)
C7—C8—C9—N1	−147.0 (2)	C21—C22—C23—C24	3.1 (5)
O2—C8—C9—C1	155.6 (3)	C21—C22—C23—Br1	−175.0 (2)
C7—C8—C9—C1	−20.9 (3)	C22—C23—C24—C25	−2.2 (4)
O2—C8—C9—C10	−85.9 (3)	Br1—C23—C24—C25	175.9 (2)
C7—C8—C9—C10	97.6 (3)	C21—C20—C25—C24	1.6 (4)
N1—C9—C10—C11	−165.7 (2)	C19—C20—C25—C24	−176.3 (2)
C1—C9—C10—C11	71.2 (3)	C23—C24—C25—C20	−0.2 (4)
C8—C9—C10—C11	−40.8 (3)	C9—N1—C26—C27	114.1 (3)
N1—C9—C10—C18	78.4 (2)	C28—N1—C26—C27	−22.5 (3)
C1—C9—C10—C18	−44.8 (3)	C9—N1—C26—C19	−8.5 (3)
C8—C9—C10—C18	−156.8 (2)	C28—N1—C26—C19	−145.1 (2)
N1—C9—C10—C19	−44.6 (2)	C20—C19—C26—N1	−150.6 (2)
C1—C9—C10—C19	−167.8 (2)	C10—C19—C26—N1	−21.3 (3)
C8—C9—C10—C19	80.2 (2)	C20—C19—C26—C27	90.6 (3)
C18—C10—C11—O3	−165.4 (3)	C10—C19—C26—C27	−140.1 (2)
C19—C10—C11—O3	−36.6 (4)	N1—C26—C27—S1	35.1 (3)
C9—C10—C11—O3	75.1 (3)	C19—C26—C27—S1	151.8 (2)
C18—C10—C11—C12	15.4 (3)	C28—S1—C27—C26	−31.1 (3)
C19—C10—C11—C12	144.1 (2)	C9—N1—C28—S1	−133.7 (2)
C9—C10—C11—C12	−104.2 (2)	C26—N1—C28—S1	−1.1 (3)
O3—C11—C12—C17	172.7 (3)	C27—S1—C28—N1	19.3 (2)

### Hydrogen-bond geometry (Å, °)

**Table:**

*D*—H···*A*	*D*—H	H···*A*	*D*···*A*	*D*—H···*A*
C18—H18B···O1	0.97	2.42	3.080 (4)	125.
C22—H22A···O2^i^	0.93	2.44	3.172 (4)	135.

Symmetry codes: (i) −*x*, −*y*+2, −*z*+2.
